# Interlink between solubility, structure, surface and thermodynamics in the ThO_2_(s, hyd)–H_2_O(l) system

**DOI:** 10.3389/fchem.2022.1042709

**Published:** 2022-11-15

**Authors:** Christian Kiefer, Thomas Neill, Nese Cevirim-Papaioannou, Dieter Schild, Xavier Gaona, Tonya Vitova, Kathy Dardenne, Jörg Rothe, Marcus Altmaier, Horst Geckeis

**Affiliations:** ^1^ Institute for Nuclear Waste Disposal, Karlsruhe Institute of Technology, Karlsruhe, Germany; ^2^ Research Centre for Radwaste Disposal and Williamson Research Centre for Molecular Environmental Science, Department of Earth and Environmental Sciences, The University of Manchester, Manchester, United Kingdom

**Keywords:** thorium, solubility, surface, temperature, thermodynamics, structure

## Abstract

The impact of temperature on a freshly precipitated ThO_2_(am, hyd) solid phase was investigated using a combination of undersaturation solubility experiments and a multi-method approach for the characterization of the solid phase. XRD and EXAFS confirm that ageing of ThO_2_(am, hyd) at *T* = 80°C promotes a significant increase of the particle size and crystallinity. TG-DTA and XPS support that the ageing process is accompanied by an important decrease in the number of hydration waters/hydroxide groups in the original amorphous Th(IV) hydrous oxide. However, while clear differences between the structure of freshly precipitated ThO_2_(am, hyd) and aged samples were observed, the characterization methods used in this work are unable to resolve clear differences between solid phases aged for different time periods or at different pH values. Solubility experiments conducted at *T* = 22°C with fresh and aged Th(IV) solid phases show a systematic decrease in the solubility of the solid phases aged at *T* = 80°C. In contrast to the observations gained by solid phase characterization, the ageing time and ageing pH significantly affect the solubility measured at *T* = 22°C. These observations can be consistently explained considering a solubility control by the outermost surface of the ThO_2_(s, hyd) solid, which cannot be properly probed by any of the techniques considered in this work. Solubility data are used to derive the thermodynamic properties (log *K°_s,0_, Δ_f_ *G*°_m_) of the investigated solid phases, and discussed in terms of particle size using the Schindler equation. These results provide new insights on the interlink between solubility, structure, surface and thermodynamics in the ThO_2_(s, hyd)–H_2_O(l) system, with special emphasis on the transformation of the amorphous hydrous/hydroxide solid phases into the thermodynamically stable crystalline oxides.

## 1 Introduction

In the case of early canister failure, actinide chemistry in the near-field of a repository for the disposal of high-level radioactive waste (HLW) will be affected by elevated temperatures of up to 200°C, depending upon host-rock system and repository concept. The corrosion of iron occurring after the closure of the repository and possible water access will promote reducing conditions, for which the oxidation states + III and +IV are expected to control the solution chemistry of the actinides, An (Grenthe et al., 2020). In aqueous systems, An (IV) behavior is dominated by the formation of sparingly soluble, nanoparticulate and amorphous hydrous oxides, AnO_2_ (am, hyd), and by a strong tendency for hydrolysis (Rand et al., 2009; Grenthe et al., 2020). The transition of these amorphous oxy-hydroxides into the thermodynamically stable crystalline oxides AnO_2_ (cr), is kinetically hindered due to their low solubility, and is generally not observed in aqueous systems. However, ageing processes induced by time or temperature may facilitate this transition with the consequent decrease of the overall solubility.

The solubility and hydrolysis of thorium have been extensively investigated in the literature, although solubility studies involving a concurrent, thorough solid phase characterization are sparse. Hietanen and co-workers conducted potentiometric and coulometric experiments with Th(IV) in 3 M NaCl solutions at 25°C (Hietanen and Sillen, 1968). Based on their own experimental data and the reinterpretation of previous studies (Hietanen, 1954; Kraus and Holmberg, 1954; Baes et al., 1965), the authors derived a speciation model involving the predominance of the polynuclear species Th_2_(OH)_2_^6+^, Th_2_(OH)_3_^5+^ and Th_6_(OH)_14_^10+^. This chemical model was updated in the review work by Baes and Mesmer, who reported the hydrolysis scheme and corresponding equilibrium constants in the reference state (*I* = 0, *T* = 25°C) including the hydrolysis species ThOH^3+^, Th(OH)_2_^2+^, Th(OH)_4_ (aq), Th_2_(OH)_2_^6+^, Th_4_(OH)_8_^8+^ and Th_6_(OH)_15_^9+^ (Baes and Mesmer, 1976). In 2001, Neck and Kim published the most comprehensive study on An (IV) solubility and hydrolysis, which included the critical review of previously reported studies and estimations of hydrolysis constants based on a semi-empirical electrostatic model. For Th(IV), the authors selected a hydrolysis scheme including the species ThOH^3+^, Th(OH)_2_^2+^, Th(OH)_3_^+^, Th(OH)_4_ (aq), Th_4_(OH)_12_^4+^ and Th_6_(OH)_15_^9+^, and reported the solubility product log *K*°_s,0_ (Th(OH)_4_, am) = –47.0 (Neck and Kim, 2001). Neck and co-workers reported also solubility experiments combined with laser-induced breakdown detection (LIBD) and X-ray absorption fine structure (XAFS) (Neck et al., 2002). The solubility product was determined to be log *K*°_s,0_ = –47.8, whereas XAFS and LIBD confirmed the presence of large amounts of small Th(IV) colloids within 3.5 < pH < 5. Altmaier et al. investigated the solubility and the colloid formation of Th(IV) in 0.5 M and 5.0 M NaCl as well as in 0.25, 2.5 and 4.5 M MgCl_2_ solutions (Altmaier et al., 2004). The authors emphasized the relevant role of intrinsic colloids in the aquatic chemistry of thorium in neutral and alkaline solutions. Kobayashi et al. conducted solubility experiments and investigated the differences on solid phases and solubility after aging a freshly precipitated Th(IV) hydroxide at 363 K for 3–6 weeks (Kobayashi et al., 2016). The authors observed a significant decrease on the solubility correlating with a slight growth of the particle size in the aged solid, for which a log *K*°_s,0_ = –51.6 was reported. Nishikawa *et al.* investigated the solubility of Th(IV) in 0.5 M NaClO_4_ and HClO_4_ solutions with 2.0 < pH < 8.0 (Nishikawa et al., 2018). The starting material Th(OH)_4_(am) was aged at 298 K, 313 K and 333 K for up to 40 weeks. Solubility measurements conducted with the aged solid phases were also conducted at 298, 313 and 333 K, and showed a systematic decrease of solubility with increase of the ageing temperature. The expected increase of crystallinity at elevated temperatures was confirmed by XRD measurements.

Most of the available solubility studies were conducted using amorphous hydrous oxides. The nomenclature used to define such solid phases includes Th(OH)_4_(am), ThO_2_⋅*x*H_2_O(am), ThO_2_(am, hyd), ThO_2_(am), among others, thus highlighting the ill-defined character of the solid phase controlling the solubility in these studies (Dzimitrowicz et al., 1985; Rai et al., 1997; Neck and Kim, 2001; Altmaier et al., 2004; Rand et al., 2009). Although the crystalline oxides AnO_2_(cr) are the thermodynamically stable An(IV) end-members (Rand et al., 2009; Grenthe et al., 2020), the amorphous hydrous oxides are actually controlling An(IV) solubility in aqueous systems and thus are considered to estimate solubility upper limits required in the safety assessment of repositories for nuclear waste disposal. The transition of An(IV) amorphous solid/colloidal phases into the thermodynamically stable AnO_2_(cr) has been correlated with the increase of the particle-size (Neck et al., 2007). However, no attempts have been made so far to link the loss of hydroxide groups/water in the transformation of AnO_2_(am, hyd) into AnO_2_(cr), or to understand how this affects the stability of the corresponding solid phases, as well as the interlink with surface area and particle size.

The structures of ThO_2_(am, hyd) nanoparticles and precursors, and AnO_2_(am, hyd) more generally, have been extensively investigated using extended X-ray absorption fine structure (EXAFS) analysis. Th coordination environments found varied from aqueous Th^4+^-like coordination for early hydrolysis products, with Th coordinated by 10–13 O backscatterers (likely H_2_O and OH^−^) at 2.44–2.51 Å and few or no Th backscatterers (Neck et al., 2002; Rothe et al., 2002), to more crystalline ThO_2_-like systems for heat treated nanoparticles, with seven to nine O backscatterers at 2.41 Å and a Th shell fit at 3.9–4.0 Å with up to 12 Th backscatterers (Bonato et al., 2020; Manaud et al., 2020). Rothe et al. analysed Th particles formed *via* hydrolysis and precipitation of colloids at pH 1.3–3.6 (Rothe et al., 2002). The resulting EXAFS fitting for the majority of samples indicated a coordination environment similar to that of aqueous Th, with only the highest pH sample and the precipitated ‘fresh’ ThO_2_(am,hyd) showing evidence of Th-Th backscatterers and long-range order. This is consistent with the findings of Neck *et al.* who, using a combination of EXAFS and laser-induced breakdown detection (LIBD), showed the structure of Th colloids formed after titration of acidic solution had high Th-O and low Th-Th coordination numbers relative to crystalline ThO_2_ (Neck et al., 2002). More recently, EXAFS studies into the structure of nanoparticulate ThO_2_ and PuO_2_ have shown that the nearest neighbor O shell alters significantly for very small particles (<10 nm) (Bonato et al., 2020). This trend was quantified by the Debye-Waller factor of the first Th-O shell, which was observed to decrease with increasing particle size (attained by TEM) and attributed to structural disorder which was more prevalent at smaller nanoparticle sizes. There was also a direct correlation observed between particle size and Th-Th coordination number; the larger the particle size, the higher the Th-Th coordination. This trend increased most significantly at small particle sizes and appeared to approach full Th-Th coordination of ThO_2_ with particles >25 nm in size. Other investigations into thermally synthesized ThO_2_ nanoparticles treated at 220-250°C found that the nanoparticles were highly crystalline and there was little or no variation in particle structure at different formation temperatures (Manaud et al., 2020). An XRD and XAFS study into ThO_2_ nanoparticles of sizes from 2.5 to 33.8 nm found that, with decreasing particle size, there was a systematic shift to larger lattice parameters by 1.1%, which coincided with a decrease in Th-Th coordination number, consistent with other studies discussed here (Plakhova et al., 2019). Amidani and co-workers investigated ThO_2_ nanoparticles by means of HEXS and HERFD XANES (Amidani et al., 2021). The authors observed mixed thorium hexamer clusters with 1 nm nanoparticles in the initial steps of formation, which lead to more crystalline, thermodynamically stable nanoparticles when exposed to elevated temperatures (150–1,000 °C). Recently, Romanchuk *et al.* compiled a systematic study of AnO_2_ (An = Th, U, Pu) and CeO_2_ nanoparticles, illustrating the similarities between the systems and suggested that the reduced coordination numbers observed in EXAFS fitting was a result of the core-shell nature of AnO_2_ nanoparticles (Romanchuk et al., 2022).

In this context, this work aims at investigating the impact of temperature, ageing time and pH on the structure and solubility of a freshly precipitated ThO_2_(am, hyd) solid phase. A multi-method approach including XRD, SEM, XPS, TG-DTA and EXAFS is used to thoroughly characterize the resulting solid phases, with special focus on particle size, degree of hydration and surface properties. In combination with solubility data, this information is used to derive thermodynamic properties for the solid phases investigated, which are compared with thermodynamic data selected in the NEA-TDB reference database. The results contribute to the quantitative description of radionuclide aqueous systems of potential relevance in the context of high-level waste disposal. At a more fundamental level, the work provides new insights on the structural evolution of the ThO_2_(s, hyd) solid phases in the transition from amorphous to crystalline, with special focus on the role of water and surface effects.

## 2 Experimental

### 2.1 Chemicals

Thorium nitrate pentahydrate (Th(NO_3_)_4_∙5H_2_O), sodium chloride (NaCl), nitric acid (ultrapure), HCl Titrisol^©^ and NaOH Titrisol^©^ were purchased from Merck. Ethanol (99.9%) was obtained from VWR Chemicals. All solutions were prepared with Milli-Q water (Milli–Q academic, Millipore, 18.2 MΩ cm). Before use, Milli-Q water was purged with Ar for >1 h to remove traces of dissolved CO_2_(g). All samples were prepared and stored in an Ar-glove box (<1 ppm O_2_), either at *T* = 22 or 80°C.

### 2.2 pH measurements

pH values were measured with combination glass electrodes (Orion ROSS), calibrated against commercial pH buffer solutions (Merck, pH 2–10). In salt solutions of ionic strength *I* ≥ 0.1 mol kg^−1^, the measured pH value (pH_exp_) is an operational value related to [H^+^] by pH_m_ = pH_exp_ + A_m_ (with [H^+^] in molal units). A_m_ is an empirical parameter accounting for the liquid junction potential of the electrode and the activity coefficient of H^+^, which is given as a function of the background electrolyte concentration. Values of A_m_ in NaCl solutions were taken from the literature (Altmaier et al., 2003).

### 2.3 Solid phase preparation and solubility experiments with ThO_2_(s, hyd)

A^232^Th(IV) stock solution was prepared by slow titration of a 0.15 M Th(NO_3_)_4_ solution to pH ≈ 10–11 using 1.0 M NaOH. The resulting solid, *i.e.* ThO_2_(am, hyd), was separated from the nitrate-rich supernatant by centrifugation at 4,000 *g* for 10–15 min. The solid phase was dissolved in 0.1 M HCl, and the procedure was repeated until nitrate was washed out (<10 ppm, determined with colorimetric test strips Merck MQuant^®^). Approximately 1.2 g of ThO_2_(am, hyd) solid phase was obtained in a final slow titration, which was divided into nine aliquots of approximately 130 mg each. Eight of the aliquots were contacted with either 0.1 M HCl–NaCl (pH_m_ ≈ 3) or 0.1 M NaOH (pH_m_ = 12.8) at *T* = (22 ± 2) °C, and aged at *T* = (80 ± 2)°C for 1, 2, 4.5 and 5.5 months. The remaining aliquot was used for the study of the freshly precipitated hydrous oxide. Due to the temperature-dependence of the pK_w_ of water, the actual pH_m_ of the 0.1 M NaOH suspensions was significantly lower at *T* = (80 ± 2) °C, *i.e.* pH_m_ (*T* = 80°C) = 11.2.

Different series of undersaturation solubility experiments at *T* = 22°C were prepared using the fresh and aged solid phases described above. Each independent batch sample was prepared with 1.5–3 ml of the corresponding solid suspension (fresh precipitate or solid phases aged at *T* = 80°C). This aliquot was centrifuged for 5 min at 4,000 *g*, separated from the supernatant, and washed two times with the corresponding equilibration solution. After the last washing step, the solid phase was contacted with 5–20 ml (depending upon pH_m_) of the equilibration solution. Seven series of solubility experiments were prepared using a freshly precipitated ThO_2_(am, hyd), and ThO_2_(s, hyd) aged for 1, 2 and 4.5 months (pH_m_ = 3 and 12.8) at *T* = (80 ± 2)°C. These solid phases were equilibrated at *T* = 22°C in 0.1 M HCl–NaCl solutions with 2.3 < pH_m_ < 6.3. Concentration of Th and pH were monitored at regular time intervals until equilibrium conditions were attained (defined by constant [Th] and pH_m_ readings). For the determination of thorium concentrations, an aliquot of the supernatant of each sample was centrifuged (12,000 *g*) with 10 kDa filters (NanoSep Merck Millipore, pore size ∼2 nm). A given volume of the resulting filtrate was diluted with 2% ultrapure HNO_3_, and Th concentration was quantified by ICP-MS (Perkin Elmer ELAN 6100).

### 2.4 Solid phase characterization

#### 2.4.1 XRD, TG-DTA, SEM and XPS

X-ray powder diffraction (XRD) measurements were performed with a Bruker AXS D8 Advance X-Ray powder diffractometer (Cu-Kα radiation, LynxEye XE-T detector). An aliquot with approximately 1–2 mg of each solid phase was washed 3 times with 0.5 ml of ethanol to remove the matrix solutions. After the last washing step, the solid phase was re-suspended in ethanol and deposited as suspension on a spot prepared with vaseline on a XRD sample plate. The measurement angle was 2° < 2θ < 70° with incremental steps of 0.015° and a measurement time of 0.4 s for each step. Diffractograms collected were compared with reference data reported in the Joint Committee on Powder Diffraction Standard (JCPDS, 2001). Based on the full width at half maximum (FWHM) of the diffraction peaks, the crystallite size for a given solid was calculated using the Scherrer equation (Scherrer, 1918; Holzwarth and Gibson, 2011).

An aliquot of the washed solid was prepared on an indium foil, dried under Ar atmosphere, and subsequently analyzed by X-ray photoelectron spectroscopy (XPS) and scanning electron microscopy (SEM). XPS measurements were performed with a XPS system PHI 5000 VersaProbe II (ULVAC-PHI Inc.) equipped with a scanning microprobe X-ray source (monochromatic Al Kα, hν = 1,486.7 eV). Binding energies of elemental lines are charge referenced to the oxidic portion of the O 1s spectrum at 530.0 eV. A FEI Quanta 650 FEG environmental scanning electron microscope (now Thermo Fisher Scientific Inc.) was used to image the carbon coated sample surfaces. The primary electron beam energy was 30 keV. Relative atomic concentrations (H not detected) were calculated by areas of elemental lines of survey spectra, recorded at 187.85 eV pass energy of the analyzer, after subtraction of a local Shirley background and taking into account sensitivity factors and asymmetry parameters of elemental lines, and the transmission function of the analyzer (Moulder et al., 1995; Briggs and Grant, 2003). Relative error of semiquantitative atomic concentrations is typically within ± (10–20)%. Curve fits to narrow scans of elemental lines recorded at 23.5 eV pass energy were performed by Gaussian functions after Shirley background subtraction. Molar concentrations of elemental species are calculated by atomic concentrations and curve fit results. Data analysis was performed using ULVAC-PHI MultiPak program, version 9.9.

Thermogravimetric analysis (TG) with differential thermal analysis (DTA) were performed under Ar atmosphere using a Netzsch STA 449C equipment. Measurements were performed with 10–20 mg of dry solid material. Samples were heated up to 1,200 °C with a heating rate of 10 K min^−1^.

#### 2.4.2 EXAFS measurements

Th L_3_ edge XAFS spectra were recorded at the INE beamline of the KIT Light Source (KARA storage ring), Karlsruhe, Germany (Rothe et al., 2019). Ge (4 2 2) crystals were used in the Lemonnier-type double crystal monochromator. The monochromated radiation was focused by a Rh-coated toroidal mirror resulting in a spot size of <1 mm at the sample position. EXAFS measurements were performed with selected solid samples, *i.e.* ThO_2_(am, hyd, fresh) and three aged solid phases ThO_2_(s, hyd, aged): *t* = 1 m at pH_m_ = 3, *t* = 1 m at pH_m_ = 12.8 and *t* = 5.5 m at pH_m_ = 12.8. Approximately 10–15 mg of each solid phase was washed once with a weakly alkaline solution (pH_m_ ≈ 9.2) and re-suspended in a small volume of the same solution. The resulting suspensions were placed into a sealed, liquid nitrogen stable sample holder. The plastic cells were contained under Ar atmosphere in a gas-tight cell and transported to the synchrotron source, where they were stored under Ar atmosphere until the EXAFS measurements. Samples were measured at 80 K in a liquid nitrogen cooled cryostat in fluorescence acquisition mode simultaneously using a 1-pixel and a 4-pixel silicon drift (Vortex) detector. The excitation energy scale was calibrated using a ThO_2_ reference standard (first inflection point calibrated to 16,300 eV). The resulting XAFS spectra were processed and analysed using Athena and Artemis software from the Demeter software package (FEFF 6) (Ravel and Newville, 2005). The spectra were fit using a ‘shell-by-shell’ approach using fixed coordination numbers to limit number of fit parameters, and the statistical validity of each shell was verified by way of an F-test (>95% validity) (Downward et al., 2007).

## 3 Results and discussion

### 3.1 Solid phase characterization

#### 3.1.1 XRD

**FIGURE 1:**
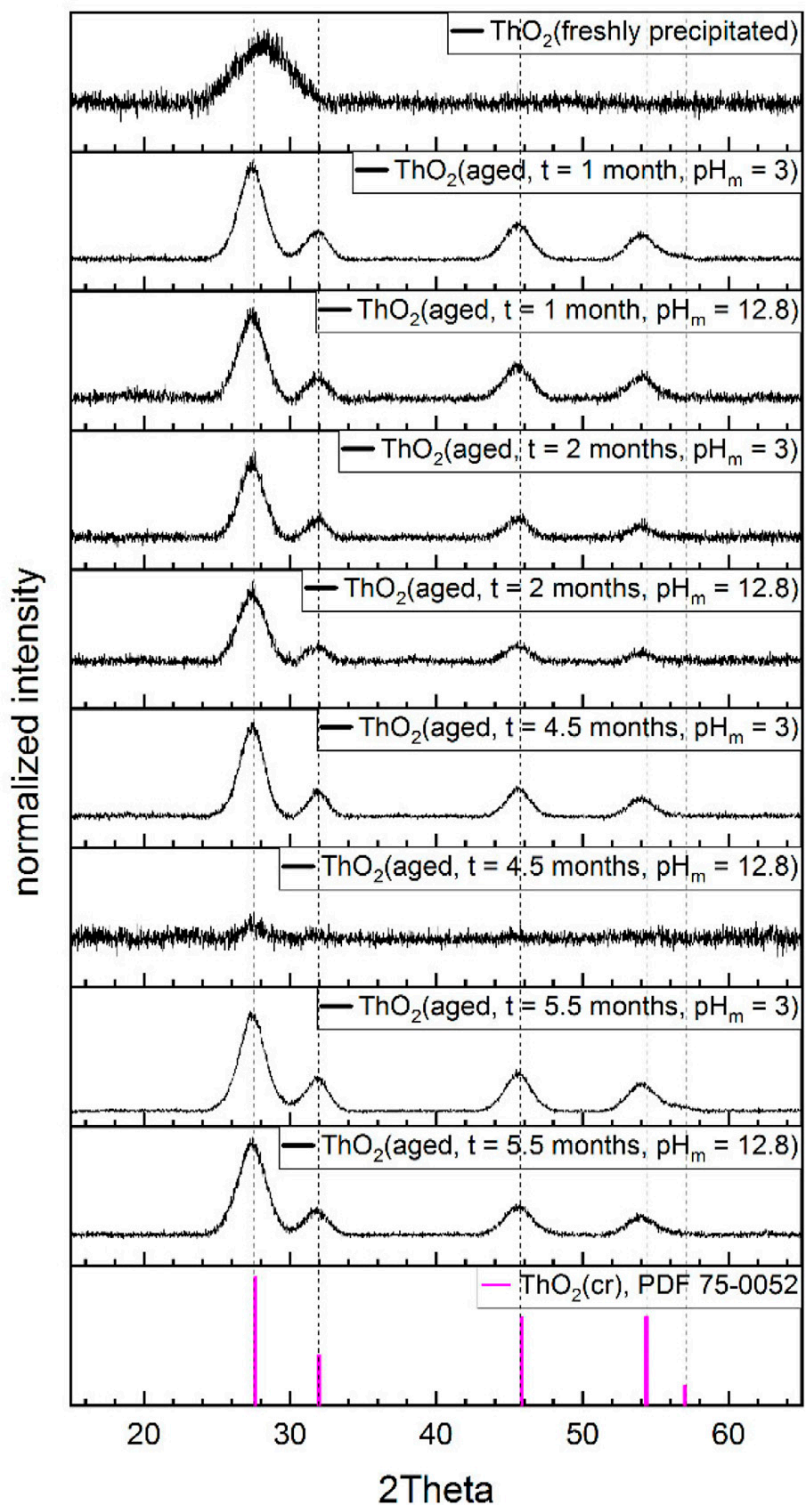
Diffractograms of the Th(IV) solid phases synthesized in this work and equilibrated at T = 80°C and pH_m_(25°C) = 3 and 12.8, except for the sample “ThO_2_(freshly precipitated)”, which was measured 2 days after precipitation. Vertical dashed lines refer to the ThO_2_(cr) reference (PDF 75-0052). The low quality of the diffractogramm obtained for the sample aged for 4.5 months at pH_m_ = 12.8 was caused by the limited amount of solid phase available.

Figure 1 shows the powder diffractograms collected for the fresh and aged Th(IV) solid phases investigated within this study. The figure includes also the main diffraction lines reported for the ThO_2_(cr) reference (PDF 75-0052).

The diffractogram obtained for the freshly precipitated material shows a main broad feature at 2Θ ≈ 28.5°, which reflects the amorphous character of the solid phase. This is in line with previous observations reported by Kobayashi and co-workers for freshly precipitated Th(IV) hydrous oxide (Kobayashi et al., 2016). In all other cases, XRD patterns show narrower peaks with well-defined 2Θ positions, reflecting a higher degree of crystallinity for the Th(IV) aged samples. No significant differences are visually observed in the diffractograms of Th(IV) solid phases aged for different times or at different pH_m_ values. The peak positions in the XRD of the aged samples are in excellent agreement with reference data reported for ThO_2_(cr) in the JCPDS database, with main reflections at 2Θ = 27.6, 32.0, 45.9, 54.4 and 57.0°. A good agreement is also obtained with the peak positions in the XRD reported by Kobayashi *et al.* for Th(IV) solid phases aged at *T* = 90°C for 3–6 weeks in 0.1–2.0 M NaClO_4_ and 0.1–3.0 M NaCl (Kobayashi et al., 2016). Plakhova and co-workers reported well-resolved XRD patterns for freshly precipitated ThO_2_(s, hyd) (Plakhova et al., 2019). However, we note that the drying process at *T* = 40 and 150°C followed by the authors may have increased the degree of crystallinity of the originally precipitated Th(IV) solid phase.

Table 1 shows the results of the Scherrer analysis based on the evaluation of the full width at half maximum (FWHM) intensity of the XRD peaks in Figure 1. Large differences are observed between the crystallite size of freshly precipitated and aged ThO_2_(s, hyd) solid phases, whereas very similar crystallite sizes are quantified for solid phases aged at *T* = 80°C for samples aged for different lengths of time. For the same ageing time, the Scherrer analysis hints towards the formation of slightly larger crystallites in the solid phases aged at pH_m_ = 3 than for those aged at pH_m_ = 12.8, although the values of the crystallite size overlap within their uncertainties. This observation could be rationalized by the higher solubility of Th(IV) at pH_m_ = 3 (≈10^−2^ M, calculated at *T* = 25°C for ThO_2_(am, hyd, aged)) than at pH_m_ = 12.8 (≈10^−8^ M), which may result in a faster particle growth through enhanced dissolution and precipitation reactions.

**TABLE 1:** Scherrer analysis of the ThO_2_(s, hyd, fresh) and ThO_2_(s, hyd, aged) solid phases investigated in this work. n.d. stands for not determined.

Sample	Crystallite size [nm]
ThO_2_(s, hyd, fresh, *T* = 22°C)	(1.5 ± 0.5)
ThO_2_(s, hyd, aged, *t* = 1 m, pH_m_ = 3, *T* = 80°C)	(4.4 ± 0.5)
ThO_2_(s, hyd, aged, *t* = 1 m, pH_m_ = 12.8, *T* = 80°C)	(4.1 ± 0.5)
ThO_2_(s, hyd, aged, *t* = 2 m, pH_m_ = 3, *T* = 80°C)	(4.7 ± 0.5)
ThO_2_(s, hyd, aged, *t* = 2 m, pH_m_ = 12.8, *T* = 80°C)	(4.1 ± 0.5)
ThO_2_(s, hyd, aged, *t* = 4.5 m, pH_m_ = 3, *T* = 80°C)	(4.8 ± 0.5)
ThO_2_(s, hyd, aged, *t* = 4.5 m, pH_m_ = 12.8, *T* = 80°C)	n.d
ThO_2_(s, hyd, aged, *t* = 5.5 m, pH_m_ = 3, *T* = 80°C)	(4.6 ± 0.5)
ThO_2_(s, hyd, aged, *t* = 5.5 m, pH_m_ = 12.8, *T* = 80°C)	(4.1 ± 0.5)

Kobayashi *et al.* reported crystallite sizes between 3.1 and 4 nm for selected Th(IV) solid phases aged at *T* = 90°C (Kobayashi et al., 2016), in moderate agreement with crystallite sizes determined in this work. Plakhova and co-workers reported crystallite sizes of ≈2 and ≈3.6 nm for ThO_2_(s, hyd) solid phases precipitated at room temperature (but dried at *T* = 40 and 150°C) in 3.0 M NH_3_⋅H_2_O and 3.0 M NaOH, respectively (Plakhova et al., 2019). The hydrothermal treatment of Th(IV) solid phases in H_2_O (*T* = 210°C, pH not specified) and 3.0 M NaOH (*T* = 180°C) resulted in particle sizes of ≈4.3 and ≈4.7 nm, respectively.

#### 3.1.2 TG-DTA, SEM and XPS

**FIGURE 2:**
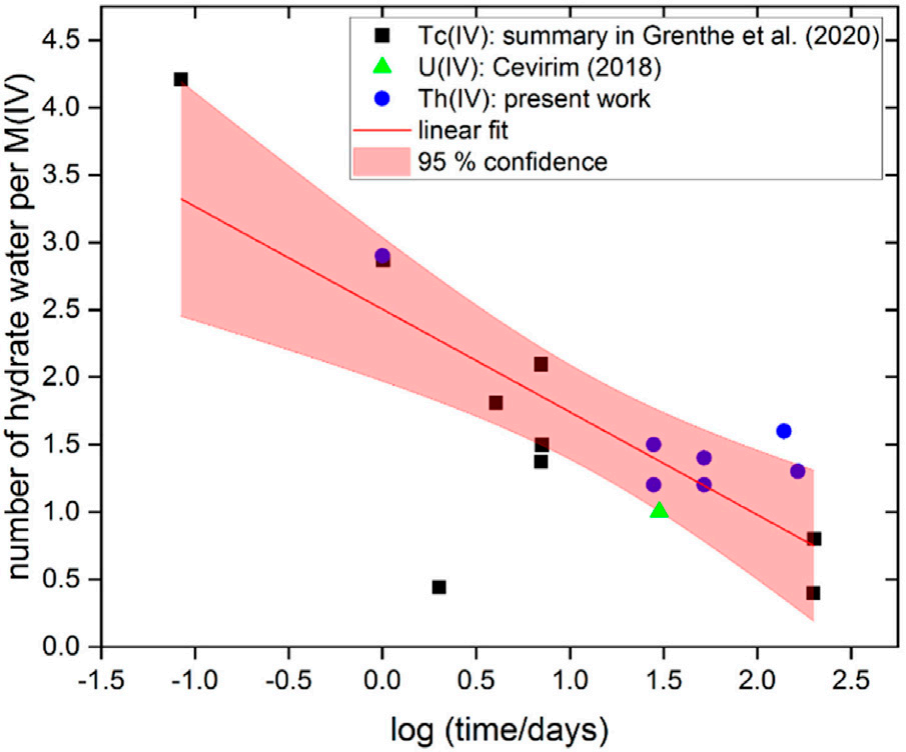
Evolution of the number of hydration waters in M(IV)O_2_ (s, hyd) with time as reported in this work for Th or reported in the literature for U (Cevirim-Papaioannou, 2018) and Tc (Yalcintas et al., 2016; Grenthe et al., 2020). Figure modified after (Grenthe et al., 2020) (Tc chapter, prepared by B. Grambow).

In the process of particle growth through ageing (either at room or elevated temperatures), a decrease of the number of hydration waters in the solid ThO_2_(am, hyd)^1^ is expected. The number of hydration waters in An (IV) hydrous oxides has been often assumed as 2 (*i.e.* AnO_2_⋅2H_2_O(s) ≅ An(OH)_4_(s)), although a few previous studies report clearly lower values of hydration waters (0.6–1) for M(IV) solid phases aged for 1–3 years at room temperature (Yalcintas et al., 2016; Cevirim-Papaioannou, 2018). TG-DTA measurements were performed to determine the number of hydration waters of freshly precipitated ThO_2_(am, hyd) as well as of solid phases aged at *T* = 80°C for *t* = 1, 2, 4.5 and 5.5 months. The main quantitative outcome evaluated from the TG-DTA data is the total weight loss measured up to 1,200°C, which is assigned to the number of hydration waters in the investigated hydrous oxides (see Table 2). This evaluation approach is a simplification of the actual situation, where loosely bound water, sticking moisture, hydroxide groups and (less likely) crystal waters might be present. The results in the table clearly show a significantly larger amount of hydration waters in the freshly precipitated solid (*n* = 2.9 ± 0.1) compared to the aged phases (*n* = 1.4 ± 0.2). No clear trend is observed for the latter as a function of time or pH. The values of hydration water determined for the aged solid phases are in line with data reported by Cevirim-Papaioannou for a U(IV) hydrous oxide phase aged at *T* = 22°C for up to 798 days (*n* = 1.0 ± 0.5) (Cevirim-Papaioannou, 2018). Figure 2 shows the evolution of the number of hydration waters in M(IV)O_2_ (s, hyd) as a function of time, with M = Th (this work), U (Cevirim-Papaioannou, 2018) and Tc (Yalcintas et al., 2016; Grenthe et al., 2020). The figure shows a clear qualitative trend to decrease the number of hydration waters with ageing time, thus supporting the transformation of hydroxide/hydrated phases into the corresponding, thermodynamically stable, oxides.

**TABLE 2:** Weight loss and calculated number of hydration waters as quantified by TG-DTA for the Th(IV) hydrous phases investigated in this work.

Sample	Weight loss	Hydration water
ThO_2_(s, hyd, fresh, *T* = 22°C)	(16.7 ± 0.5) %	(2.9 ± 0.1)
ThO_2_(s, hyd, aged, *t* = 1 m, pH_m_ = 3, *T* = 80°C)	(9.1 ± 0.5) %	(1.5 ± 0.1)
ThO_2_(s, hyd, aged, *t* = 1 m, pH_m_ = 12.8, *T* = 80°C)	(7.7 ± 0.5) %	(1.2 ± 0.1)
ThO_2_(s, hyd, aged, *t* = 2 m, pH_m_ = 3, *T* = 80°C)	(7.6 ± 0.5) %	(1.2 ± 0.1)
ThO_2_(s, hyd, aged, *t* = 2 m, pH_m_ = 12.8, *T* = 80°C)	(8.8 ± 0.5) %	(1.4 ± 0.1)
ThO_2_(s, hyd, aged, *t* = 4.5 m, pH_m_ = 3, *T* = 80°C)	(9.9 ± 0.5) %	(1.6 ± 0.1)
ThO_2_(s, hyd, aged, *t* = 5.5 m, pH_m_ = 12.8, *T* = 80°C)	(8.0 ± 0.5) %	(1.3 ± 0.1)

All SEM pictures show irregular aggregates of ≈20 to ≈100 nm (see Figure SI-1 in Supporting Information). No clear trend can be observed as function of the ageing time or ageing pH, which is likely related to the very small and similar particle size observed for all aged samples. Similar aggregates were previously reported for other An(IV)O_2_(am, hyd) systems (with M = Tc, U, Np, Pu) precipitated at room temperature (Fellhauer, 2013; Yalcintas, 2015; Cevirim-Papaioannou, 2018). Note that much more regular aggregates were obtained in recent studies investigating the size and local environment of Th(IV) nanoparticles exposed to much higher temperatures (400–1,000°C) (Amidani et al., 2019; Bonato et al., 2020).

**FIGURE 3:**
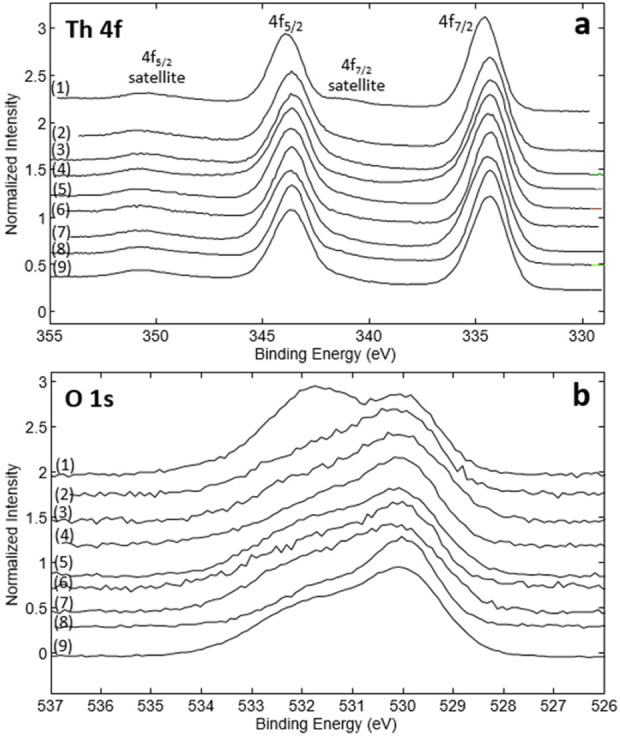
XPS narrow scans of Th(IV) hydrous oxides: **(A)** Th 4f lines; **(B)** O 1s lines. (1) freshly precipitated solid phase. Solids aged at T = 80°C: (2) 1 month at pH_m_ = 3; (3) 1 month at pH_m_ = 12.8; (4) 2 months at pH_m_ = 3; (5) 2 months at pH_m_ = 12.8; (6) 4.5 months at pH_m_ = 3; (7) 4.5 months at pH_m_ = 12.8; (8) 5.5 months at pH_m_ = 3; (9) 5.5 months at pH_m_ = 12.8.

Supplementary Figures SI-2A–C in the Supporting Information exemplarily shows the complete survey XP spectrum of the Th(IV) sample equilibrated at *T* = 80°C for 2 months at pH_m_ = 3, as well as the narrow scans of the Th 4f and the O 1s lines. The narrow scan of the O 1s line includes also the fit with the hydrate, hydroxide and oxide contributions. The XP spectra of all investigated solids are shown together in Figures 3A,B. Table 3 summarizes the atom % of Th and O as well as the ratio [O]/[Th] quantified considering the intensities of the Th 4f and the O 1s lines. In all cases, the ratio O: Th is well above 2 (2.3–2.8), thus underpinning the significant presence of hydroxide and hydrate groups that enhance the ratio [O]/[Th] beyond the value of two present in the crystalline ThO_2_(cr). Table 3 summarizes also the speciation of oxygen (as hydrate, hydroxide or oxide) resulting from the fit of the O 1s line.

**TABLE 3:** Atomic % of Th and O, ratio [O]/[Th] and speciation of oxygen (as atomic percent of oxide, hydroxide and water) as calculated from the Th 4f and O 1s lines in the XPS measurements. Relative error of atomic concentrations is within ± (10–20) %.

Solid phase	Atom % Th	Atom % O	Ratio [O]/[Th]	O^2-^ (%)	OH^−^ (%)	H_2_O (%)
ThO_2_(s, hyd, fresh, *T* = 22°C)	27.6	72.4	2.6	43.3	47.9	8.8
ThO_2_(s, hyd, aged, *t* = 1 m, pH_m_ = 3, *T* = 80°C)	30.4	69.6	2.3	56.1	30.5	13.4
ThO_2_(s, hyd, aged, *t* = 1 m, pH_m_ = 12.8, *T* = 80°C)	28.7	71.3	2.5	63.5	29.8	6.7
ThO_2_(s, hyd, aged, *t* = 2 m, pH_m_ = 3, *T* = 80°C)	29.7	70.3	2.4	59.3	31.6	9.1
ThO_2_(s, hyd, aged, *t* = 2 m, pH_m_ = 12.8, *T* = 80°C)	26.1	73.9	2.8	57.0	33.8	9.2
ThO_2_(s, hyd, aged, *t* = 4.5 m, pH_m_ = 3, *T* = 80°C)	28.0	72.0	2.6	55.6	30.8	13.6
ThO_2_(s, hyd, aged, *t* = 4.5 m, pH_m_ = 12.8, *T* = 80°C)	29.3	70.7	2.4	55.6	26.9	17.5
ThO_2_(s, hyd, aged, *t* = 5.5 m, pH_m_ = 3, *T* = 80°C)	29.0	71.0	2.5	64.0	28.9	7.1
ThO_2_(s, hyd, aged, *t* = 5.5 m, pH_m_ = 12.8, *T* = 80°C)	28.6	71.4	2.5	58.4	31.6	10.0

Consistently with other solid phase characterization techniques considered in this work (XRD, TG-DTA), XPS provides clear evidence of the differences between the freshly precipitated Th(IV) hydrous oxide and the corresponding solids aged at *T* = 80°C. However, no clear trends are observed in the XPS analysis of solid phases aged for different times or at different pH values. The fraction of oxide calculated as average of all solid phases equilibrated at pH_m_ = 3 (58.8 ± 5.8%) is virtually the same as the average obtained for solid phases equilibrated at pH_m_ = 12.8 (58.6 ± 4.9%). However, the oxide content in all aged samples is clearly above the fraction of oxide quantified for the freshly precipitated Th(IV) hydrous oxide (43.3%).

Both bulk (XRD, TG-DTA) and surface-sensitive techniques (XPS) considered for the characterization of the solid phases show no clear effect of ageing time or pH on the properties of the investigated Th(IV) hydrous oxides. However, it is important to bear in mind that XPS provides an information depth of about 3 nm at the experimental conditions. Alterations affecting a monolayer at the Th(IV) oxide surface will most likely not be clearly identified by this technique.

#### 3.1.3 EXAFS

**FIGURE 4:**
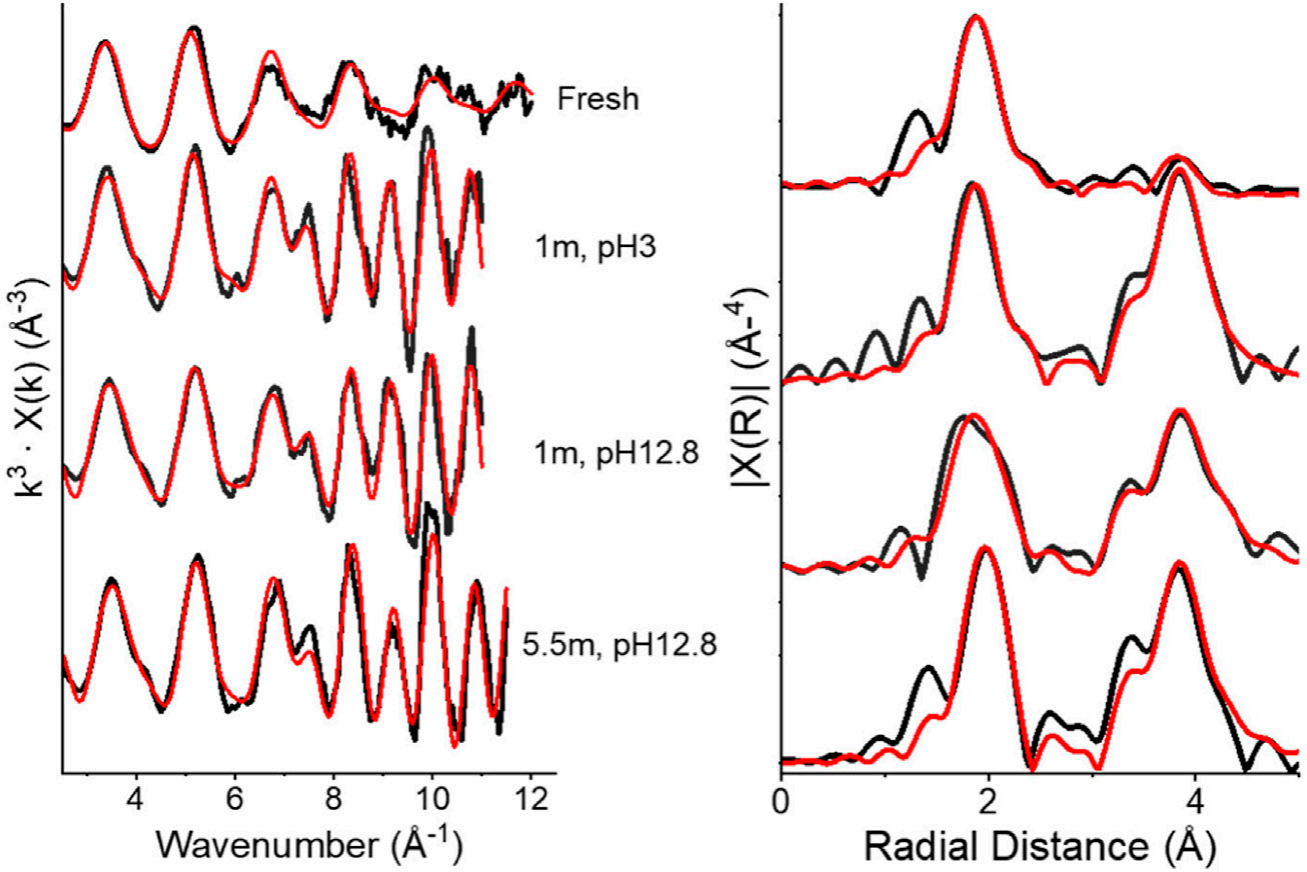
EXAFS (left) and Fourier-Transformed EXAFS (right) for (from top to bottom) ThO_2_(s, hyd) freshly precipitated, ThO_2_(s, hyd) aged, t = 1 m, pH_m_ = 3, ThO_2_(s, hyd) aged, t = 1 m, pH_m_ = 12.8 and ThO_2_(s, hyd) aged, t = 5.5 m, pH_m_ = 12.8. Black lines are experimental data and red lines are fits.

Th L_3_ edge EXAFS data and fits are shown in Figure 4, and details of best fits are presented in Table 4. Freshly precipitated as well as solid phases aged at *T* = 80 °C for 1 month (pH_m_ = 3 and 12.8) and 5.5 months (pH = 12.8) were analysed. All samples were fit with one or two O shells at 2.37–2.54 Å and a Th backscatterer at (3.96 ± 0.04) Å, with all three aged samples also including a distant O shell at (4.58 ± 0.04) Å. These distances are in general agreement with the structure of crystalline ThO_2_ (Wyckoff, 1963), however there are some deviations from the crystalline structure which are more prominent in the less aged samples. For the fresh and 1 month aged samples, at both pH_m_ 3 and 12.8, best fits contain a split first shell coordination. Here, two O shells at (2.37 ± 0.03) and (2.53 ± 0.04) Å were fit, compared to one O shell at 2.43 Å that would be expected for crystalline ThO_2_. The two shells have coordination numbers between 4.8 and three each, with total coordination ranging from 7.8 to 8.7 for each fit, which is in close agreement with the coordination number of eight for the single O shell in crystalline ThO_2_. These findings are consistent with previous EXAFS fitting of ThO_2_(am,hyd) nanoparticles (Neck et al., 2002), and this is representative of a distortion of the crystal structure in the fresh and 1 month aged precipitates. However, this distortion does not appear to affect the longer distance backscatterers for all fits, with Th and O backscatterers fit at distances anticipated for a ThO_2_-like, fluorite-type structure. The main difference between the fits is in the fresh sample, where the best fit includes only 5.5 Th backscatterers, as opposed to 12 Th backscatterers anticipated for crystalline ThO_2_. This value is much closer for the sample aged 1 month at pH = 3 (11.6), 1 month at pH = 12.8 (12) and 5.5 months at pH = 12.8 (11.4) fits. These results are consistent with the fresh sample having significantly different structure to the other three samples, likely due to a much smaller crystallite size and/or much higher structural disorder resulting in reduced long-range coordination, seen as a reduced Th backscatterer coordination number.

**TABLE 4:** EXAFS fit data for select solid samples.

Solid phase	Path	N	R (Å)	σ^2^	ΔE_0_	R
ThO_2_(s, hyd, fresh, *T =* 22°C)	Th-O_1_	4.8	2.38 (2)	0.004 (3)	4.8 (10)	0.015
	Th-O_2_	3.0	2.54 (3)	0.004 (3)	4.8 (10)	
	Th-Th	5.5	3.95 (3)	0.015 (5)	4.8 (10)	
ThO_2_(s, hyd, aged, *t* = 1 m, pH_m_ = 3, *T =* 80°C)	Th-O_1_	4.8	2.37 (3)	0.003 (1)	6.7 (15)	0.020
	Th-O_2_	3.4	2.53 (4)	0.003 (1)	6.7 (15)	
	Th-Th	11.6	3.96 (1)	0.005 (3)	6.7 (15)	
	Th-O_3_	15.0	4.58 (4)	0.008 (1)	6.7 (15)	
ThO_2_(s, hyd, aged, *t* = 1 m, pH_m_ = 12.8, *T =* 80°C)	Th-O_1_	4.7	2.37 (2)	0.004 (2)	8.1 (11)	0.016
	Th-O_2_	4.0	2.53 (2)	0.006 (3)	8.1 (11)	
	Th-Th	12.0	3.97 (1)	0.005 (1)	8.1 (11)	
	Th-O_3_	20.0	4.58 (2)	0.004 (0)	8.1 (11)	
ThO_2_(s, hyd, aged, *t* = 4.5 m, pH_m_ = 12.8, *T =* 80°C)	Th-O_1_	6.8	2.43 (1)	0.006 (2)	9.9 (10)	0.020
	Th-Th	11.4	3.96 (1)	0.005 (1)	9.9 (10)	
	Th-O_2_	17.3	4.56 (2)	0.007 (1)	9.9 (10)	
ThO_2_(cr) (Rothe et al., 2002)	Th-O_1_	8.0	2.41 (1)	0.0054	2.3	0.0241
	Th-Th	12	3.98 (2)	0.0042	4.7	
	Th-O_2_	24	4.63 (1)	0.0064	6.4	

Coordination numbers (N), U bond distances (R (Å)), Debye-Waller factors (σ^2^), shift in energy from calculated Fermi level (ΔE0) and ‘goodness of fit’ factor (R). Coordination numbers were fixed. Numbers in parentheses are the standard deviation on the last decimal place.

Overall, EXAFS fitting suggests that the structure of these nanoparticles becomes more crystalline over time. The fresh sample has the lowest Th backscatterer coordination number and a split first O shell, the two 1 month aged samples retain this split O shell, but have Th backscatterer coordination numbers more closely matching ThO_2_ and also a distant O shell at (4.58 ± 0.04) Å. Finally, the 5.5 months aged sample has a single O shell with a Th-O distance identical to that of crystalline ThO_2_, as well as Th and distant O backscatterers present with coordination numbers similar to those in the crystalline structure (Table 1, see data reported by Rothe et al., 2002).

### 3.2 Solubility experiments

**FIGURE 5:**
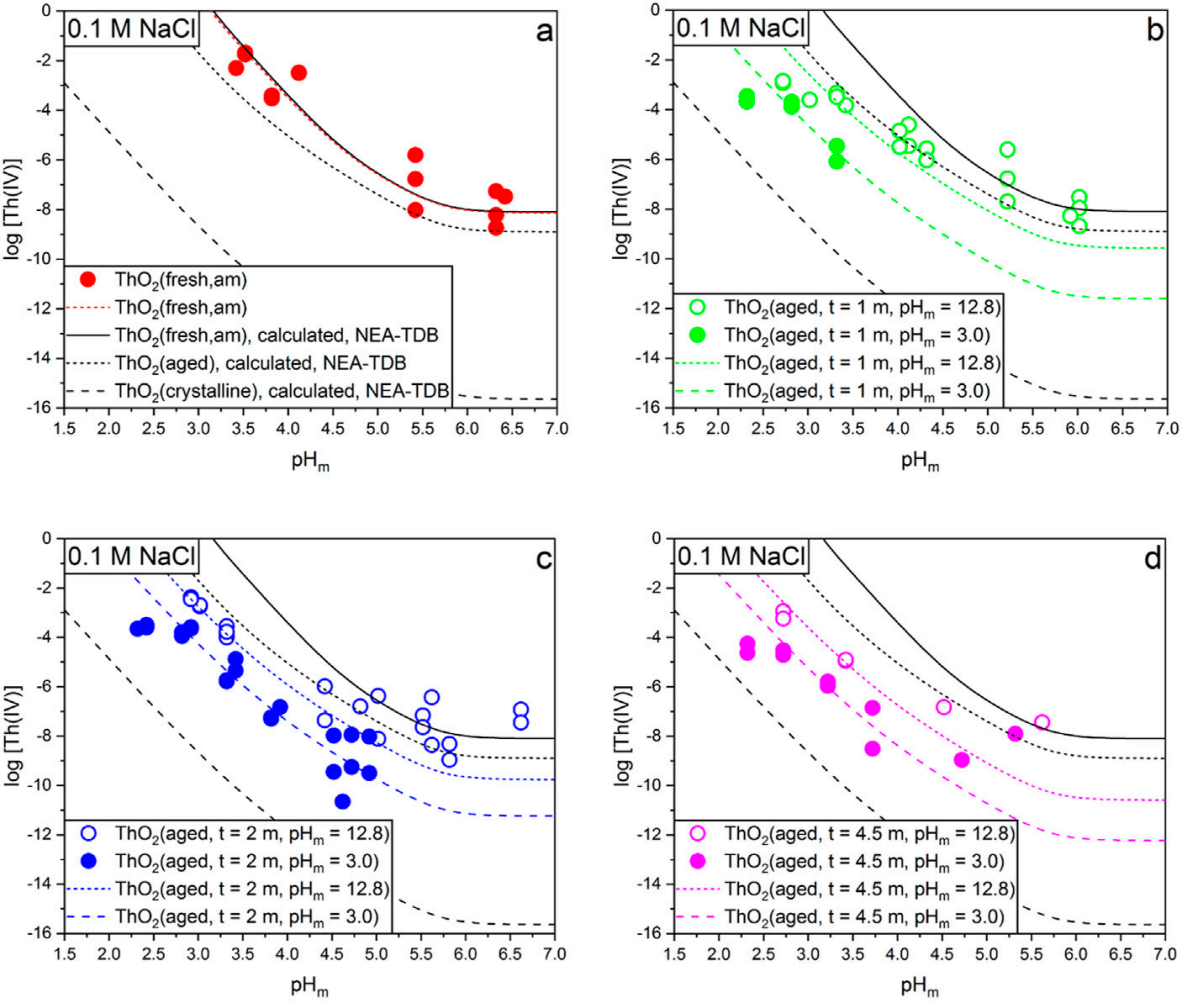
Experimental solubility data obtained in this work and model calculations using thermodynamic data derived in this work or reported in the NEA-TDB for Th(IV) hydrous oxide: **(A)** freshly precipitated; **(B)** aged for 1m at T = 80°C and pH_m_ = 3 and 12.8; **(C)** aged for 2m at T = 80°C and pH_m_ = 3 and 12.8; **(D)** aged for 4.5m at T = 80°C and pH_m_ = 3 and 12.8.

Figures 5A–D shows solubility of Th(IV) determined in this work for freshly precipitated ThO_2_(am, hyd, fresh) and ThO_2_(s, hyd, aged) aged at *T* = 80 °C for *t* = 1, 2, 4.5 m and pH_m_ = 3, 12.8. The figure also shows the solubility curves corresponding to freshly precipitated, aged and crystalline ThO_2_ solid phases calculated using the solubility and hydrolysis constants selected in the NEA-TDB (Rand et al., 2009), as well as solubility curves calculated with the solubility constants derived in this work (see Section 3.3).

The experimental data on the freshly precipitated Th(IV) hydrous oxide agrees well with the solubility calculated for ThO_2_(am, hyd, fresh) using the NEA-TDB thermodynamic selection (Figure 5A). Experimental data are also in moderate agreement with previous studies reporting the solubility of ThO_2_(am, hyd) at *T* = 25°C in 0.1 M NaCl or NaClO_4_ (Ryan and Rai, 1987; Rai et al., 2000). All aged Th(IV) solid phases show lower solubilities than ThO_2_(am, hyd, fresh), consistent with the increase in particle size/crystallinity observed by XRD. This observation supports also that aged solids are not a mixture of (nano-)crystalline and amorphous phases, as in this case the solubility should be defined by the most soluble solid, *i.e.* the amorphous phase. Solubility data obtained for the Th(IV) solid phase aged for 2 months at pH_m_ = 3 is also in good agreement with experimental data reported by Kobayashi and co-workers for a solid phase aged at *T* = 90°C during 6–8 weeks. Note however that Kobayashi *et al.* followed a different ageing approach as used in this study–each independent solubility sample was aged at *T* = 90°C at the target pH (ranging between ≈1.5 and ≈9), whereas in the present work the solid phase used in each solubility series was aged at a single pH. Because of the impact of pH in the ageing process (see next paragraph and Figure 5), the different ageing approach followed in Kobayashi *et al.* and in this work may lead to differences in the solubility data, especially in the less acidic samples. Recently, Nisbet and co-workers investigated the solubility of ThO_2_(cr) in the temperature range 150–250°C (Nisbet et al., 2018). The authors did not report solubility constants for the investigated systems, but the measured Th concentrations are clearly lower as those observed in the present study, in line with the crystalline character of their solid phase.

Experimental data in Figure 5 show that the solubility of the Th(IV) hydrous oxide slightly decreases with the ageing time at *T* = 80°C. Unexpectedly, the pH in which the Th(IV) solid phase was aged has a very significant effect on the solubility measured at *T* = 22°C. Hence, the solid phases aged at pH_m_ = 3 show up to two orders of magnitude lower solubility than the solid phases aged at pH_m_ = 12.8. This effect is reproduced for the solid phases aged during 1, 2 and 4.5 months. These observations are apparently in contradiction with the minor differences observed by XRD, TG-DTA and XPS for Th(IV) solid phases aged for different contact times and at different pH values. However, these results can be rationalized by a solubility control established by a few monolayers of the ThO_2_(s, hyd) surface. Such few monolayers have a minor weight in bulk characterization methods (XRD, TG-DTA) but also in “surface-sensitive” methods like XPS, which provides average values of a ≈3 nm layer. This hypothesis is also in line with previous studies by Grambow, Vandenborre and co-workers (Vandenborre et al., 2010; Grambow et al., 2017), who claimed that solubility measurements of ZrO_2_(s), ThO_2_(s) and UO_2_(s) are not representative of the bulk phase, but are rather controlled by surface processes of a few monolayers of the corresponding oxide. Although the starting materials used in these studies were crystalline oxides sintered at very high temperatures (400–1,000°C), the authors claimed that “solubility” of Th(IV) system was controlled by “ThO_*x*_ (OH)_*y*_ (H_2_O)_*z*_” present in the grain boundaries.

As already discussed above, the in general higher solubility of Th(IV) at lower pH induces faster recrystallization rates, concomitant with somewhat larger diameters of crystalline units. Even though the characterization methods applied in the present work are not able to resolve variations in the first surface monolayers, we assume that the higher recrystallization rate at low pH may also have an impact on the structure of surfacial ThO_*x*_ (OH)_*y*_ (H_2_O)_*z*_ species finally leading to a reduced solubility. Moreover, differences in the surface charge of the ThO_2_(s, hyd) (positive at pH_m_ = 3, negative at pH_m_ = 12.8) can possibly affect to the ageing process, and consequently influence solubility.

### 3.3 Thermodynamic evaluation of solubility phenomena

Experimental solubility data were modelled with the aim of determining the solubility constants (log *K°_s,0_) of the investigated Th(IV) hydrous oxides. The model of the system controlling the solubility of Th(IV) includes both monomeric (Th^4+^, ThOH^3+^, Th(OH)_2_^2+^, Th(OH)_4_ (aq)) and polyatomic (Th_2_(OH)_2_^6+^, Th_2_(OH)_3_^5+^, Th_4_(OH)_8_^8+^, Th_4_(OH)_12_^4+^, Th_6_(OH)_15_^9+^, Th_6_(OH)_14_^10+^) aqueous species as selected in the NEA-TDB (Rand et al., 2009), as well as the solid phases ThO_2_(am, hyd, fresh) and ThO_2_(ncr, hyd, t, pH_m_), with *t* = 1, 2, 4.5months and pH_m_ = 3, 12.8. The term “ncr” indicates the nanocrystalline character of the Th(IV) hydrous oxides solid phases obtained after the hydrothermal ageing at *T* = 80°C. The modelling approach is based on the fit of log *K_s,0_°, whereas the values of log *β_(n,m)_° are kept constant as selected by NEA-TDB. The datasets collected for each solid phase are fitted individually by minimizing the function ∑((log[Th]_exp_–log[Th]_calc_)^2^)^1/2^. The value [Th]_calc_ is the sum of the species [Th^4+^] [ThOH^3+^] [Th(OH)_2_^2+^] [Th(OH)_4_ (aq)] [Th_2_(OH)_2_^6+^] [Th_2_(OH)_3_^5+^] [Th_4_(OH)_8_^8+^] [Th_4_(OH)_12_^4+^] [Th_6_(OH)_15_^9+^] and [Th_6_(OH)_14_^10+^], and can be calculated as:

[Th]calc=K *s,0° γH+4 mH+4 aw−(2+x)+∑β *(n.m)° K *s,0°n γH+4n−m mH+4n−m awm−n(2+x) (1)

where the values of *β°_(n,m)_ are know from the NEA-TDB and *γ*_H_^+^ is calculated using the SIT formalism (Ciavatta, 1980; Grenthe et al., 2020). The values of hydration water *x* = 2.9 and *x* = 1.4 have been considered for the modelling of the freshly precipitated and aged solid phases, respectively, where *x* = (1.4 ± 0.3) is the average value of the number of hydration waters quantified for all aged solid phases. The impact of *x* in the current modelling calculations is however very minor in the conditions of this study, because the water activity in 0.1 M NaCl is close to unity, *i.e. a*_w_ = 0.9966. The outcome of this modelling exercise is shown in Figure 5, whereas Table 5 summarizes the log *K°_s,0_ values determined for all investigated Th(IV) hydrous oxides as compared to the values selected by NEA-TDB.

**TABLE 5:** Solubility constants for ThO_2_(s, hyd) and ThO_2_(cr) as determined in this work or selected in the NEA-TDB (Rand et al., 2009). The values of log *K°_s,0_ and log K°_s,0_ correspond to the equilibrium reactions ThO_2_⋅nH_2_O(s) + 4 H^+^ ⇔ Th^4+^ + (2 + *n*) H_2_O(l) and ThO_2_⋅nH_2_O(s) + (2–n) H_2_O(l) ⇔ Th^4+^ + 4 OH^−^, respectively.

Solid phase	log *K°_s,0_	log *K*°_s,0_	Source
ThO_2_(am, hyd, fresh, *T* = 22°C)	(9.3 ± 0.4)	–(46.7 ± 0.4)	present work
ThO_2_(am, hyd, fresh)	(9.3 ± 0.9)	–(46.7 ± 0.9)	NEA-TDB
ThO_2_(am, hyd, aged)	(8.5 ± 0.9)	–(47.5 ± 0.9)	NEA-TDB
ThO_2_(ncr, hyd, *t* = 1 m, pH_m_ = 3, *T =* 80°C)	(5.8 ± 0.2)	–(50.2 ± 0.2)	present work
ThO_2_(ncr, hyd, *t* = 2 m, pH_m_ = 3, *T =* 80°C)	(6.2 ± 0.4)	–(49.8 ± 0.4)	present work
ThO_2_(ncr, hyd, *t* = 4.5 m, pH_m_ = 3, *T =* 80°C)	(5.2 ± 0.5)	–(50.8 ± 0.5)	present work
ThO_2_(ncr, hyd, *t* = 1 m, pH_m_ = 12.8, *T =* 80°C)	(7.8 ± 0.5)	–(48.2 ± 0.5)	present work
ThO_2_(ncr, hyd, *t* = 2 m, pH_m_ = 12.8, *T =* 80°C)	(7.6 ± 0.1)	–(48.4 ± 0.1)	present work
ThO_2_(ncr, hyd, *t* = 4.5 m, pH_m_ = 12.8, *T =* 80°C)	(6.8 ± 0.4)	–(49.2 ± 0.4)	present work
ThO_2_(crystalline)	(1.76 ± 1.11)	–(54.24 ± 1.11)	NEA-TDB

The solubility constant determined in this work for ThO_2_(am, hyd, fresh) (log *K*°_s,0_ = 46.7 ± 0.4) is in excellent agreement with the value selected in the NEA-TDB, log *K*°_s,0_ = (46.7 ± 0.9). This supports the validity of the solubility experiments and modelling approach, and represents a sound anchoring point for the quantification of the solubility constants of the aged phases investigated in this work. A very significant decrease in the solubility constant (≥1.5 log_10_-units) with respect to the freshly precipitated solid phase is observed for the aged solid phases already after 1 month of ageing time. A further decrease in the values of log *K*_s,0_° is observed with the increase of aging period. However, the most significant effect on the values of log *K*°_s,0_ is observed as a function of the ageing pH. Hence, in average, the samples aged at pH_m_ = 3 have a solubility constant of *ca.* 2 log_10_-units lower than the solid phases aged at pH_m_ = 12.8. This behavior is a strong evidence of the effect of pH on the recrystallization rate, which is higher at pH_m_ = 3 due to the higher solubility of Th(IV) under acidic conditions.

The strong impact of the ageing time and pH on the value of log *K*°_s,0_ is however not reflected in differences observed by the different solid phase characterization techniques considered in this work, *i.e.* XRD, TG-DTA, SEM, XPS and XAFS. As discussed in Section 3.2, this likely reflects that solubility phenomena in the investigated system is mostly controlled by surface processes occurring in the first monolayer of the Th(IV) oxide (0.2–0.4 nm). Indirect evidences pointing to the same hypothesis were reported by Vandenborre and co-workers on the basis of isotopic exchange experiments with ^229^Th (Vandenborre et al., 2010; Grambow et al., 2017).

Differences in the solubility of ThO_2_(am, hyd) solid phases have been often attributed to the effect of particle size (and by the extension of the surface area) in the Gibbs energy of formation (Δ_f_ *G*°_m_) of the solid (Neck et al., 2007; Kobayashi et al., 2016). The Schindler equation correlates the Δ_f_ *G*°_m_ of an amorphous/colloidal solid phase with the Δ_f_ *G*°_m_ of the corresponding crystalline phase considering a term with the surface contribution (Schindler, 1967; Neck et al., 2007):

$$\Delta_{f}G{{}^{\circ}}_{m}\left({ThO}_{2}\left(am/col\right)\right)=\Delta_{f}G_{m}^{{}^{\circ}}\left({ThO}_{2}\left(cr\right)\right)+2/3\gamma S$$ (2)

where Δ_f_ *G*°_m_ is directly related to the solubility constant through the Gibbs energy of reaction (Δ_r_ *G*°_m_), *S* is the molar surface that depends on the particle size, and *γ* is the mean free surface energy per unit surface area of solid-liquid interface. The molar surface is defined as $S=\frac{M\alpha }{\rho d}$ where *M* is the molecular weight, *ρ* is the density of the solid, d is the particle size and *α* is a geometrical shape factor, *α* ≈ 6 for spherical particles (Neck et al., 2007). According to Schindler, *γ* can be estimated as (Schindler, 1967):

$$\gamma =-\frac{3RTlnK_{sp}^{{}^{\circ}}\left(S\to 0\right)}{2N_{A}\sum 4\pi {r_{i}}^{2}}$$ (3)

With the known data for ThO_2_(cr) (Δ_f_ *G* ^°^_m_ = –1,169.2 kJ mol^−1^, *M* = 264 g mol^−1^, *ρ* = 10.0 g cm^−3^, $\log K_{sp}^{{}^{\circ}}$ = –54.2, $r_{{Th}^{4+}}=0.105\;nm\;and\;r_{O^{2-}}=0.140\;nm$ ) the dependence of Δ_f_ *G* ^°^_m_ on the particle size is given as (Schindler, 1967; Neck et al., 2007):

$$\Delta_{f}G_{m}^{{}^{\circ}}\left({ThO}_{2},\;particle\;size\;d\right)=\left(–1169.2+129/d\left(nm\right)\right)\;kJ\;{mol}^{-1}$$ (4)

The Schindler equation acknowledges the key role of the surface on the overall stability of a solid phase, and thus on its solubility constant. Although the assumption of homogeneous spherical particles is not confirmed in the present work, experimental solubility constants determined in this study in combination with particle size determined by the Scherrer equation have been compared with model predictions using the Schindler equation. The molar standard Gibbs energy values for ThO_2_ solid phases of the present work are calculated according to the following equations:

$$\ln K_{sp}^{{}^{\circ}}=-\frac{\Delta_{R}G_{m}\left(T\right)}{RT}$$ (5)

$$\Delta_{R}G_{m}\left(T\right)=\sum \Delta_{f}G_{m}\left(Products\right)-\sum \Delta_{f}G_{m}\left(Educts\right)$$ (6)

**FIGURE 6:**
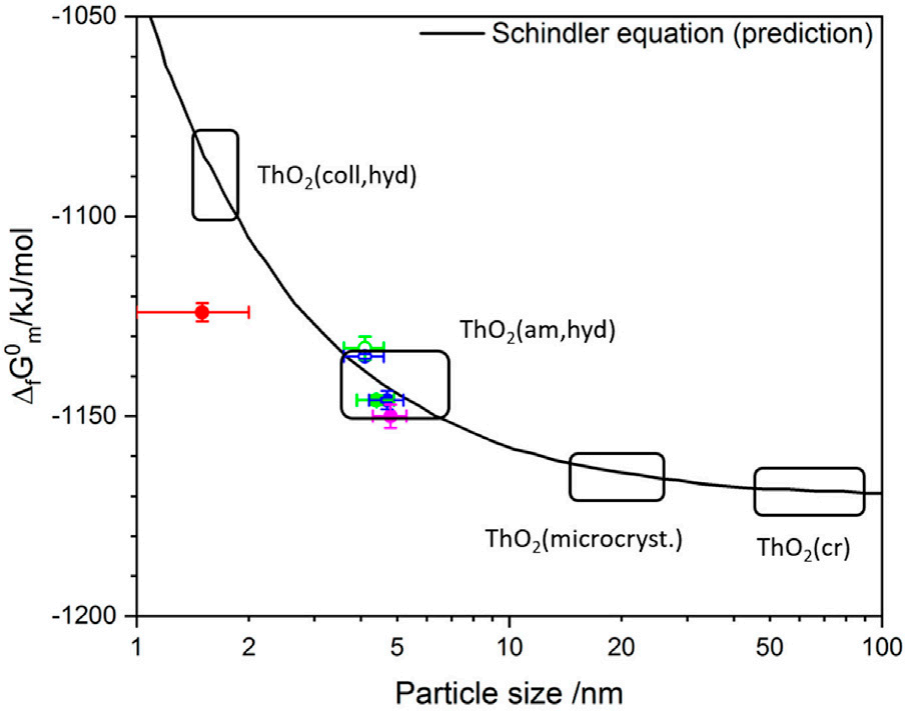
Application of Schindler equation to the ThO_2_(s) system and comparison with experimental data determined in the present work. Figure modified from (Neck et al., 2007).

In order to avoid the bias introduced by the contribution of Δ_f_ *G* ^°^_m_ (H_2_O, l) in Th(IV) oxide phases with different number of hydration waters, the following reaction is considered for the calculation of the Gibbs energy of formation of the solid:

$${ThO}_{2}(s)+4H^{+}⇌{Th}^{4+}+2H_{2}O(l)$$ (7)

which leads to:

ΔfGm°(ThO2)=RT ln⁡K *s,0°+ΔfGm°(Th4+)+2 ΔfGm°(H2O)−4 ΔfGm°(H+) (8)

where K *s,0° are the solubility constants calculated using the model derived in this study. The values of Δ_f_ *G*°_m_ for Th^4+^, H_2_O(l) and H^+^ were taken from the Th book of the NEA-TDB (Rand et al., 2009). The comparison of experimental data with predictions using the Schindler equation is shown in Figure 6.

Figure 6 shows qualitatively a good agreement between Δ_f_ *G*°_m_ calculated from experimental solubility data and model predictions using the Schindler equation. A somehow large deviation is observed for the freshly precipitated Th(IV) hydrous oxide, whereas Δ_f_ *G*°_m_ determined for the aged phases agree well with model calculations. Experimental evidences obtained in this work provide an indirect link between solubility phenomena in the ThO_2_(s) system with surface properties of a (few) monolayer(s) of the solid. Although surface is unequivocally playing a key role in the solubility equilibrium, it is less evident that particle size is the driving force controlling solubility in the ThO_2_(s) as assumed in the Schindler equation.

## 4 Summary and conclusion

The influence of temperature on Th(IV) solid phases and solubility was systematically investigated under Ar atmosphere using a freshly precipitated ThO_2_(am, hyd) solid phase as starting material. The fresh precipitate was aged at *T* = 80°C for different time periods (*t* = 1, 2, 4.5, 5.5 m) and at two different pH_m_ values (pH_m_ = 3.0, 12.8). The resulting solid phases were comprehensively characterized using a multimethod approach including XRD, TG-DTA, SEM, XPS and XAFS techniques. Selected solid phases were used for the preparation of solubility experiments at *T* = 22°C in 0.1 M NaCl solutions with 2.3 ≤ pH_m_ ≤ 7.0. These data, in combination with the hydrolysis constants and SIT coefficients selected in the NEA-TDB, were used to derive the corresponding solubility constants (log *K*°_s,0_) and assess the systematic variations caused by the ageing at elevated temperatures.

The ageing of a freshly precipitated ThO_2_(am, hyd) solid phase at *T* = 80°C induces a significant increase of the crystallinity and particle size, as confirmed by XRD measurements. TG-DTA and XPS support that this process is accompanied by an important decrease in the number of hydration waters/hydroxide groups in the original amorphous Th(IV) hydrous oxide, ThO_*x*_ (OH)_*y*_⋅*z*H_2_O(s). EXAFS data show a decreased number of Th-Th backscatterers at ≈ 3.96 Å in the freshly precipitated solid phase, however in the solid phases aged at *T* = 80°C the number of Th-Th backscatterers approaches the ThO_2_(cr) bulk value. Nevertheless, all solid characterization methods used in this work are unable to resolve clear differences between solid phases aged for different time periods or at different pH_m_ values.

Solubility experiments with fresh and aged Th(IV) solid phases conducted at *T* = 22°C show a clear decrease in the solubility of the solid phases aged at *T* = 80°C. In contrast to the observations gained by solid phase characterization, the ageing time and (specially) ageing pH_m_ are shown to have a very important impact on the solubility measured at *T* = 22°C. These apparently discordant observations can be explained in a consistent manner by claiming a solubility control by a few monolayers in the surface of the ThO_2_(s, hyd) solid (0.2–0.4 nm), which cannot be properly probed by any of the bulk (XRD, TG-DTA, SEM, XAFS) or surface-sensitive (XPS, with a penetration depth of *ca.* 4 nm) techniques considered in this work.

## Data Availability

The raw data supporting the conclusion of this article will be made available by the authors, without undue reservation.

## Footnotes

1 The most correct definition of the solid refers to ThO_*x*_(OH)_*y*_⋅zH_2_O(s), with 2*x* + *y* = 4.
