# Supplementary material for: Interlink between solubility, structure, surface and thermodynamics in the ThO2(s, hyd)–H2O(l) system
Source: Front Chem. 2022 Nov 15;10:1042709. doi: 10.3389/fchem.2022.1042709 (PMC9705364; doi:10.3389/fchem.2022.1042709)
Supplement: Supplementary file 1 [file DataSheet1.PDF]

*Supporting Information for*

# Interlink between solubility, structure, surface and thermodynamics in the $ThO_2$(s, hyd)–$H_2O$(l) system

**Christian Kiefer[1,*], Thomas Neill[1,2], Nese Cevirim-Papaioannou[1], Dieter Schild[1], Xavier Gaona[1,*], Tonya Vitova[1], Kathy Dardenne[1], Jörg Rothe[1], Marcus Altmaier[1], Horst Geckeis[1]**

[1]Institute for Nuclear Waste Disposal, Karlsruhe Institute of Technology, Karlsruhe, Germany.

[2]Research Centre for Radwaste Disposal and Williamson Research Centre for Molecular Environmental Science, Department of Earth and Environmental Sciences, The University of Manchester, Manchester, UK

*** Correspondence:**
Corresponding Authors
C. Kiefer: kiefer@subatech.in2p3.fr; X. Gaona: xavier.gaona@kit.edu

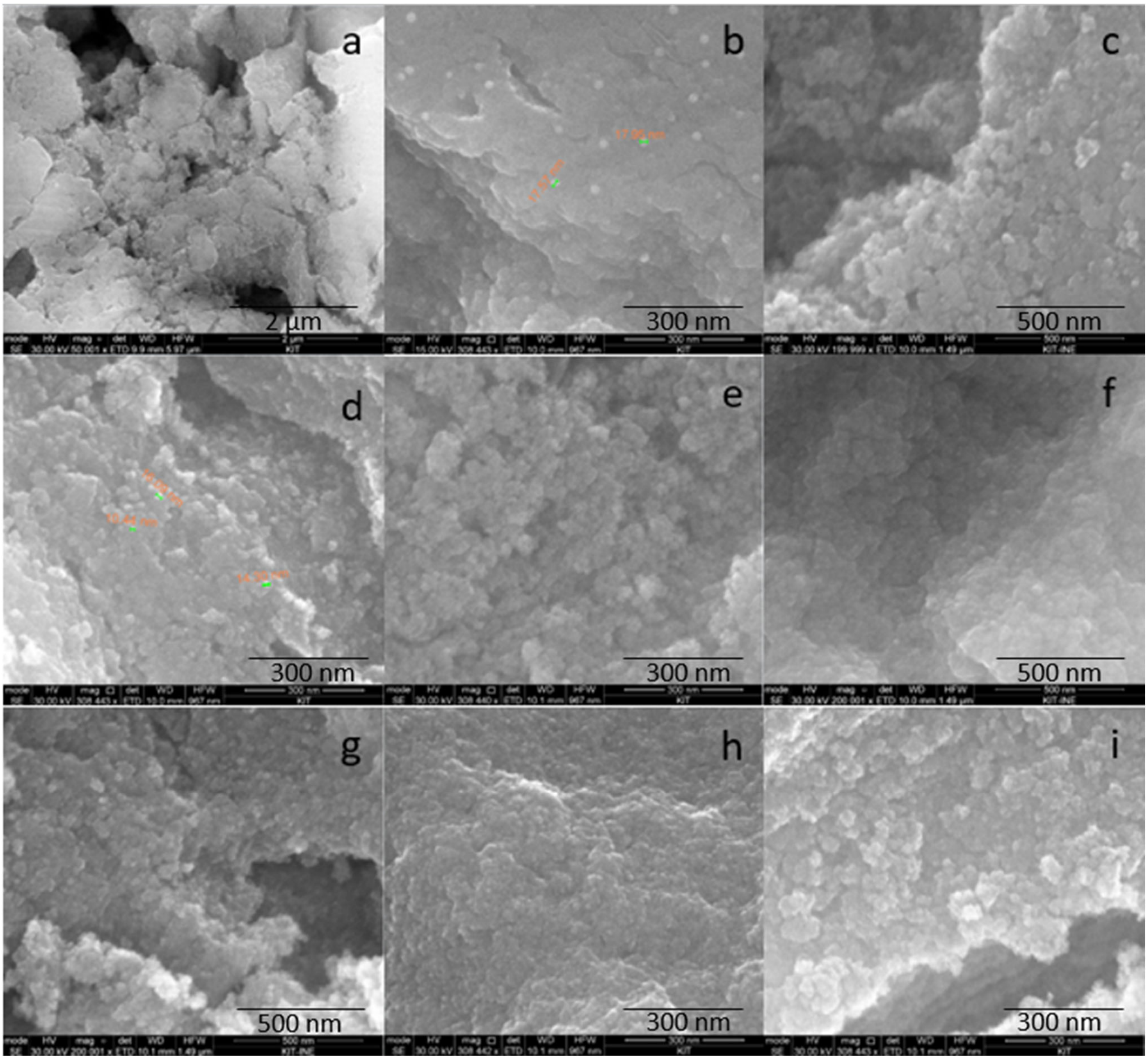


***Figure SI-1.*** *SEM images of the solid phases: (a) freshly precipitated; (b, c) aged during 1 month at* $pH_m = 3$ *and* $pH_m = 12.8$*; (d, e) aged during 2 months at* $pH_m = 3$ *and* $pH_m = 12.8$*; (f, g) aged during 4.5 months at* $pH_m = 3$ *and* $pH_m = 12.8$*; (h, i) aged during 5.5 months at* $pH_m = 3$ *and* $pH_m = 12.8$*.*

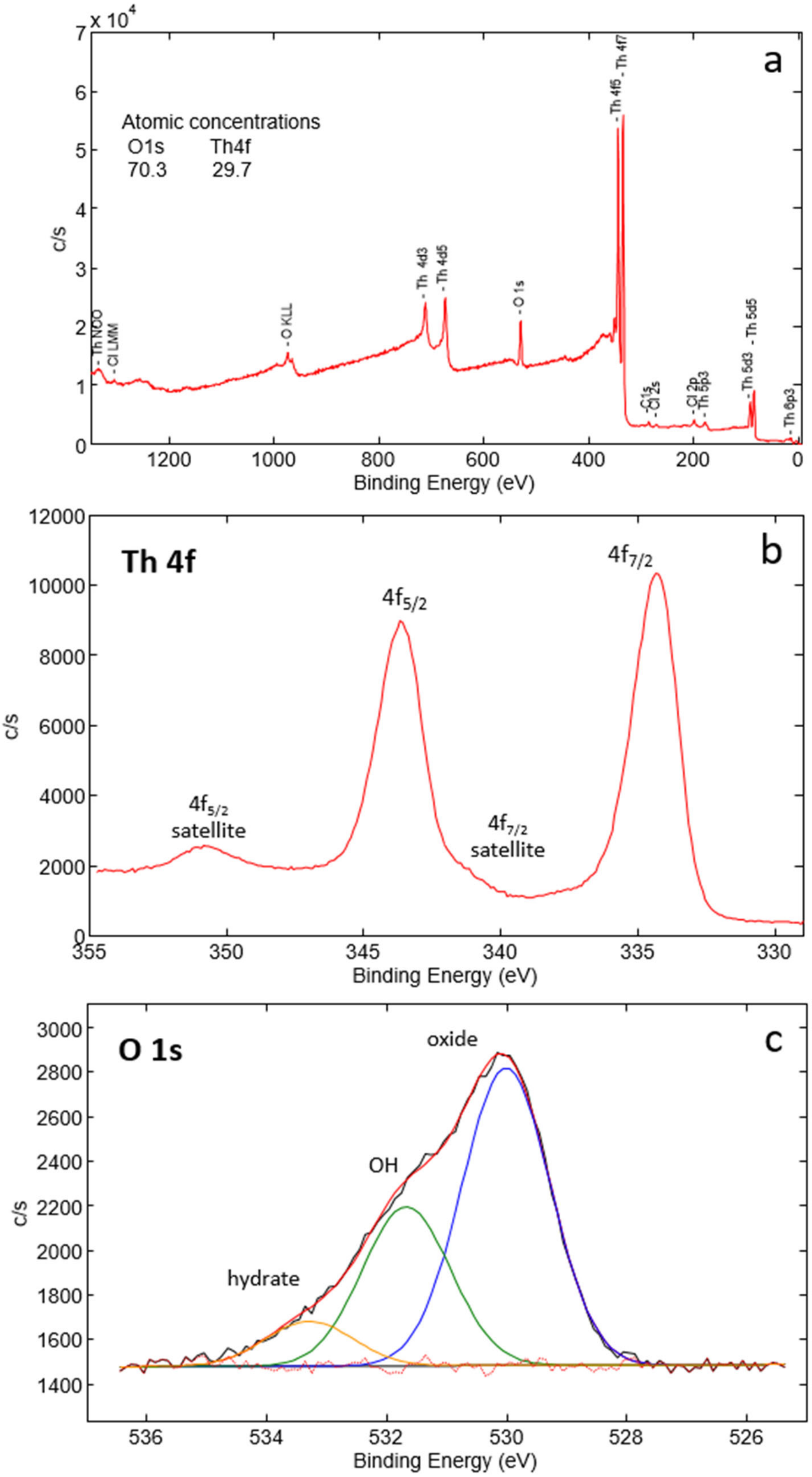


***Figure SI-2.*** *XPS spectrum of the Th(IV) hydrous oxide aged for 2 months at T = 80 °C and $pH_m$ = 3: (a) complete spectrum collected for 0-1300 eV; (b) narrow scan of the Th 4f line (c) narrow scan of the O 1s line including the fit with the hydrate, hydroxide and oxide groups.*

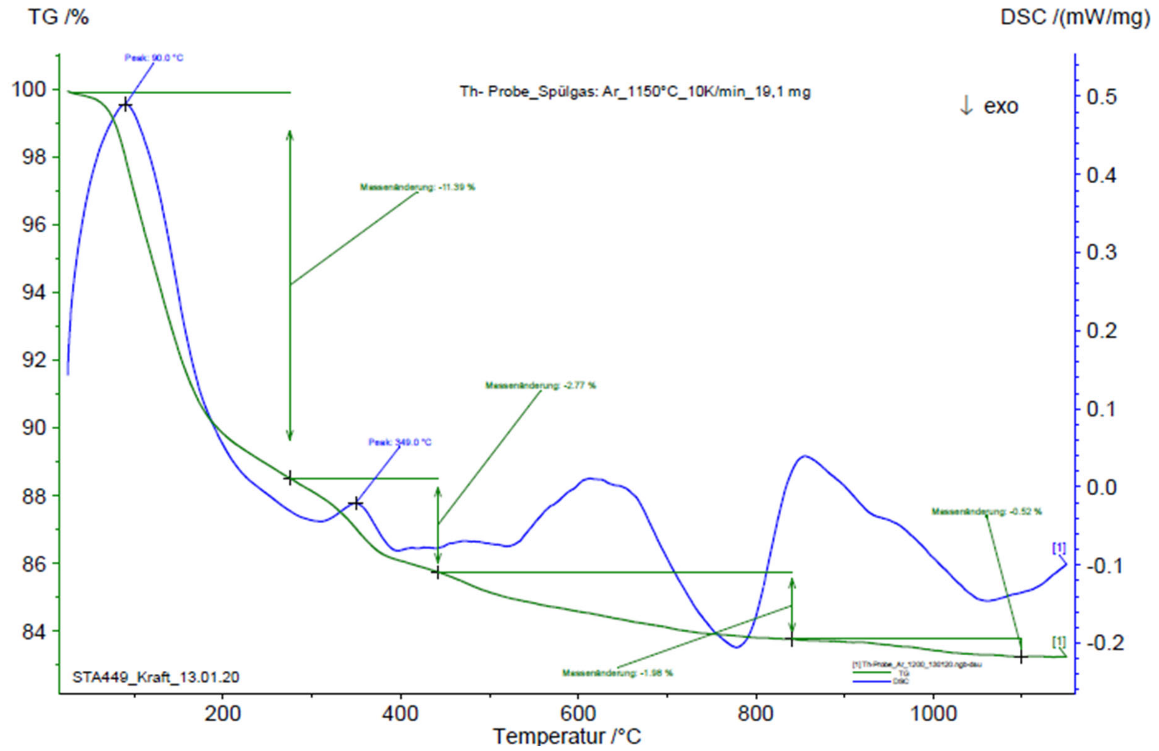


***Figure SI-3a:*** *TG-DTA results for freshly precipitated thorium dioxide solid phase.*

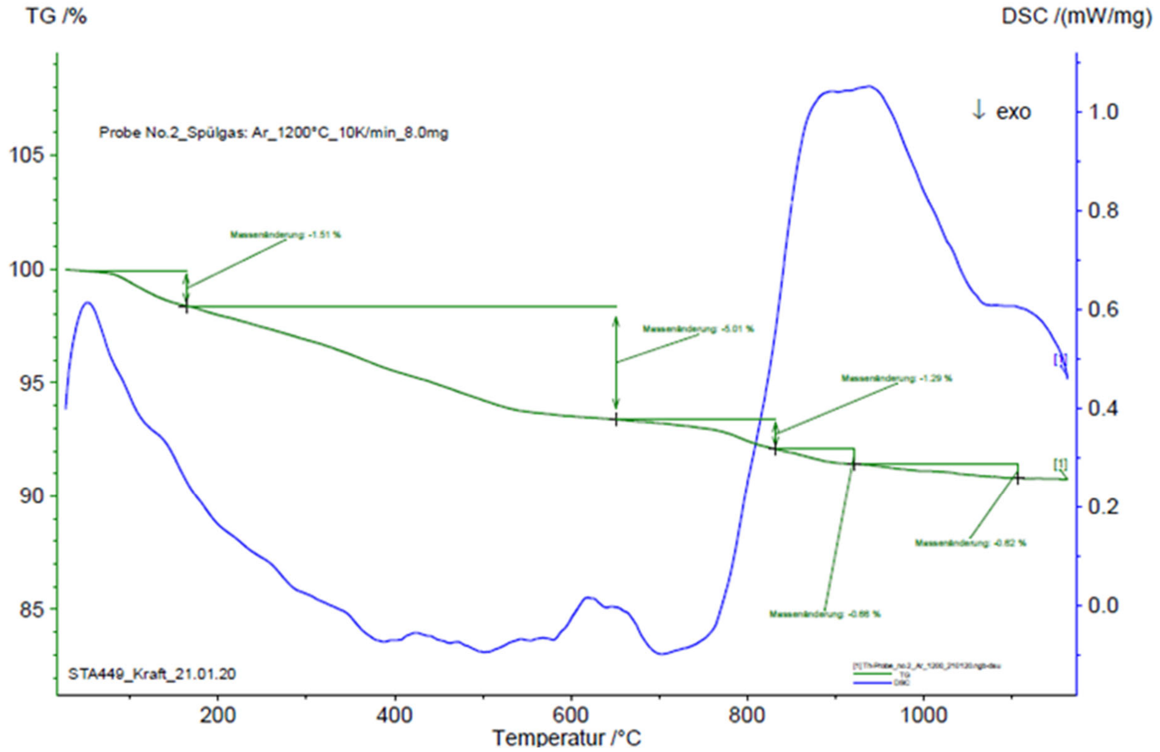


***Figure SI-3b:*** *TG-DTA results for the solid phase aged during 1 month at* $pH_m = 3$.

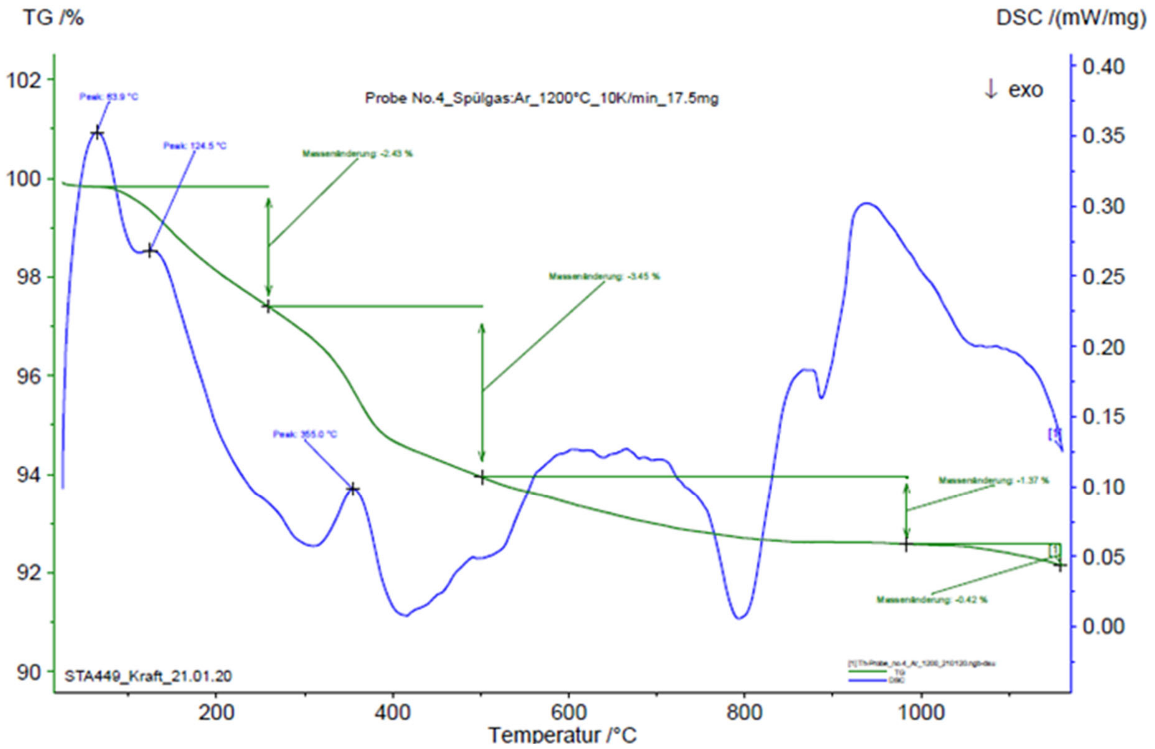


***Figure SI-3c:*** *TG-DTA results for the solid phase aged during 1 month at* $pH_m = 12.8$.

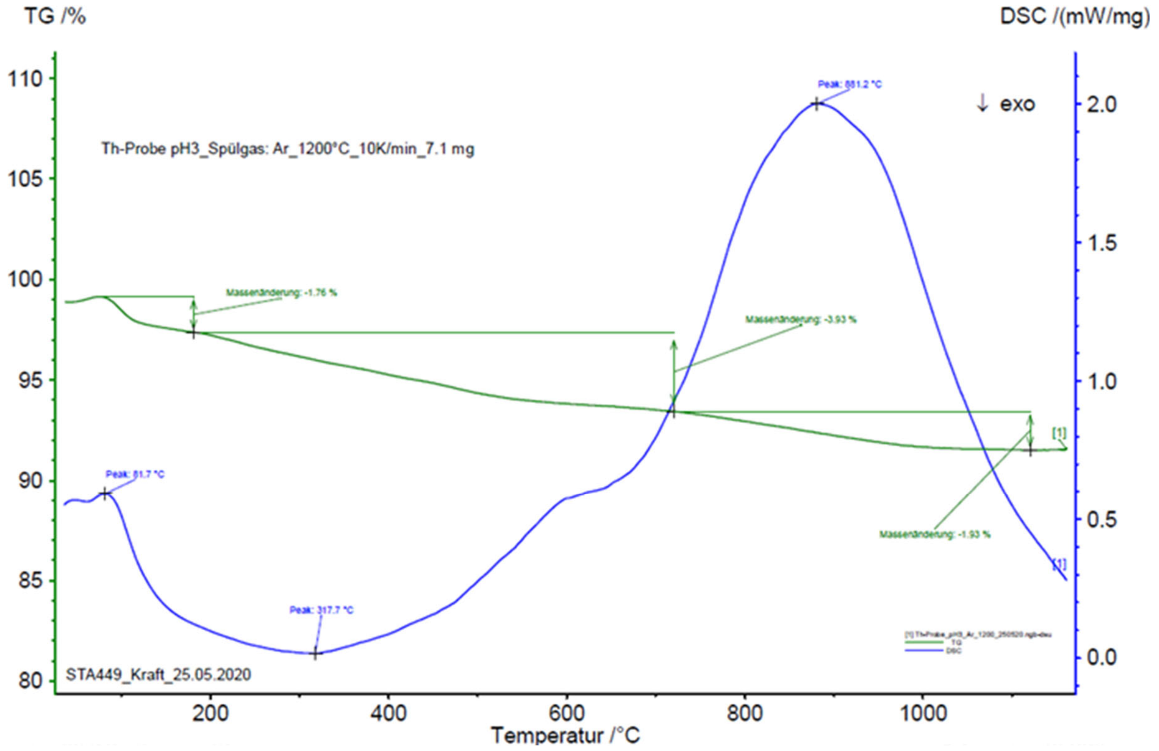


***Figure SI-3d:*** *TG-DTA results for the solid phase aged during 2 months at* $pH_m = 3$.

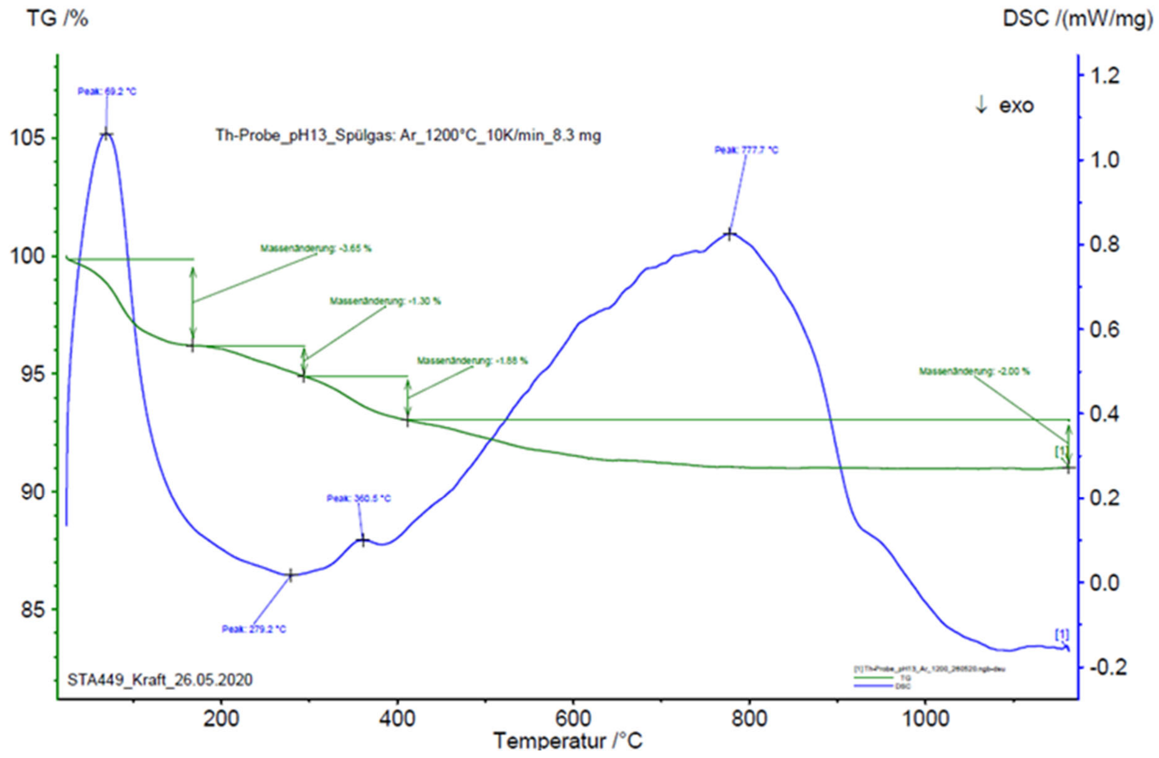


***Figure SI-3e:*** *TG-DTA results for the solid phase aged during 2 months at $pH_m$ = 12.8.*

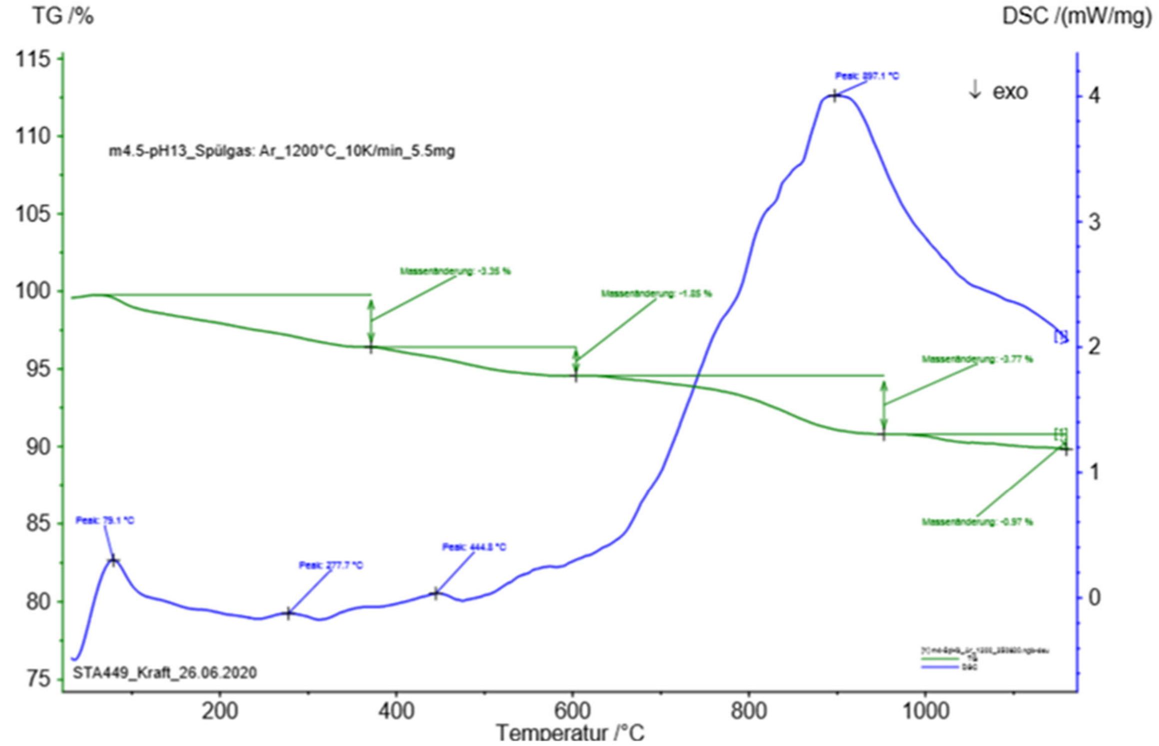


***Figure SI-3f:*** *TG-DTA results for the solid phase aged during 4.5 months at $pH_m$ = 3.*

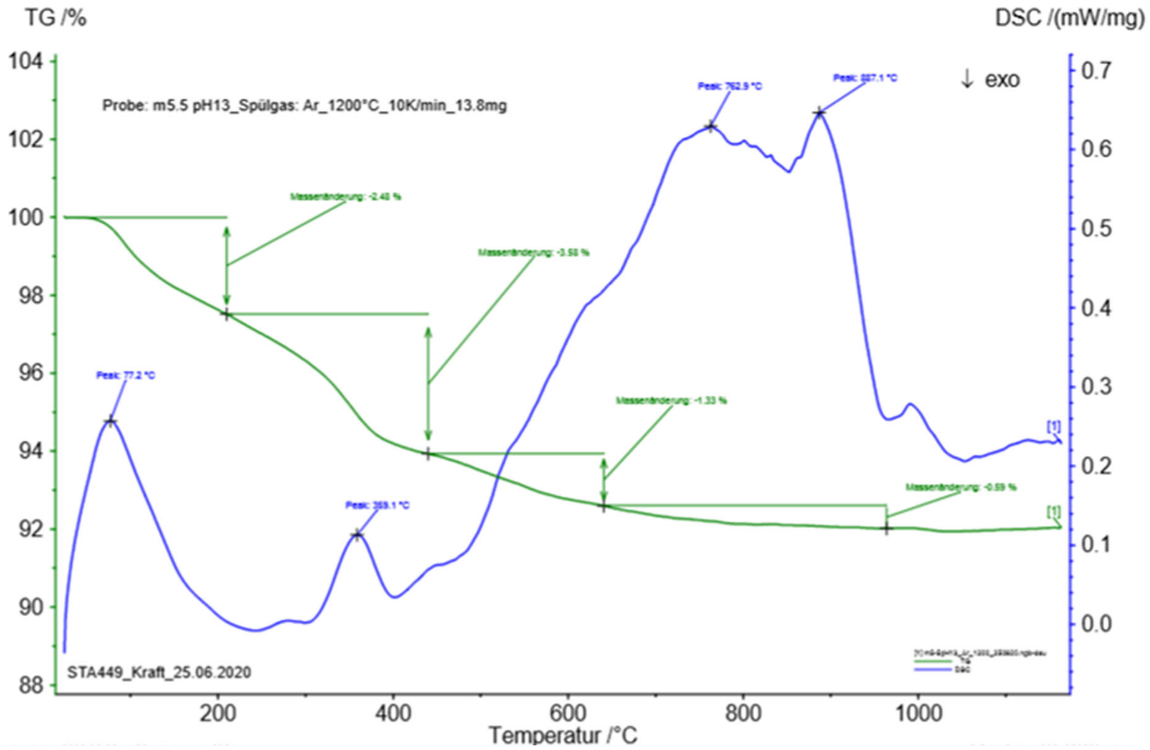


***Figure SI-3g:*** *TG-DTA results for the solid phase aged during 5.5 months at* $pH_m = 12.8$.
